# Axitinib as a third or further line of treatment in renal cancer: a single institution experience

**DOI:** 10.1186/s12894-020-00618-1

**Published:** 2020-06-02

**Authors:** G. Tsironis, M. Liontos, A. Kyriazoglou, K. Koutsoukos, A. Tsiara, M. Kaparelou, R. Zakopoulou, A. Cohen, E. Skafida, S. Fontara, F. Zagouri, A. Bamias, M. A. Dimopoulos

**Affiliations:** 1 Oncology Unit, Department of Clinical Therapeutics, Alexandra Hospital, Athens, Greece; 2 1st Department of Radiology, Aretaieio University hospital, Athens, Greece

**Keywords:** Axitinib;beyond second line, mRCC, Long responders, TKIs

## Abstract

**Background:**

Kidney cancer is a lethal neoplasm that affects several thousands of people every year. Renal cell carcinoma (RCC) is the most common histologic type. Recent developments in the therapeutic approach include antiangiogenic targeted approaches and Immunotherapy. Thus, the therapeutic algorithm of RCC patients and the survival outcomes have changed dramatically.

**Methods:**

Herein we present a retrospective study of the patients treated in our Department with an antiangiogenic agent -Axitinib, a tyrosine kinase inhibitor- as a third or further line treatment. Statistical analysis was performed with SPSS, including the available clinicopathological data of the patients included.

**Results:**

Axitinib was found to be active in patients who received this treatment beyond second line. The toxicity profile of this regimen did not reveal any unknown adverse events.

**Conclusions:**

Our real world data reflect that axitinib is a safe and effective option, even beyond the second line.

## Background

Kidney cancer represents the ninth most common cancer with an estimation of 63.990 new cases and 14.400 deaths in US for 2017 [1]. Renal cancer is most often diagnosed in males than in females and the median age at diagnosis is 64 years [1]. Five year survival estimates about 74% for all stages ranging from 12% for patients with distant metastasis to 93% for patients with localized disease [2]. Renal cell carcinoma is the most common histological type of kidney cancer, accounting for about 85–90% of cases, whereas clear cell carcinoma (80%), papillary (10–15%) and chromophobe (4–5%) are its three most common subtypes [3, 4].

Antiangiogenetic agents [5–9] along with mTOR inhibitors [10, 11] or their combination [12] and immune checkpoint inhibitors [13], led to the regulatory approval of 10 different agents for metastatic RCC patients the last 12 years resulting in a significant increase of overall survival of RCC patients [14, 15]. Available treatments have not only increased survival of patients, but also improved their quality of life and performance status allowing more lines of therapy [16]. The latter also correlates with improved survival [16]. The plethora of available treatment options is depicted to the European Association of Urology guidelines (EAU) which include Immunotherapy, antiangiogenic agents and mTOR inhibitors [17].

Little data exist though to guide therapy decisions beyond 2nd line and are limited to the results of GOLD trial, a subanalysis of RECORD-1 trial and subgroups of patients in the cabozatinib and nivolumab studies [9, 13, 18, 19]. This field is further complicated by the recent approval of immunotherapy/VEGF-TKIs combination as frontline treatment. As a result, it is very common in everyday clinical practice “first and second line” agents that have not been given before to be used beyond second line.

Axitinib is a selective inhibitor of VEGF receptors 1,2 and 3 that has been approved in patients with mRCC progressing after sunitinib or cytokines as a result of AXIS trial [20]. This second generation TKI was found superior to sorafenib in terms of PFS regardless of prior treatment (6.7 months vs 4.7 months, HR: 0.665; 95% CI 0.544–0.812; one-sided *p* < 0.0001), in partial responses (19.4 vs. 9.4%, *p* < 0.001) whereas in OS did not reach significance (mOS 20.1 months, 95% CI 16.7–23.4 vs 19.2 months 17.5–22.3, HR: 0.969, 95% CI 0.800–1.174; one-sided *p* = 0.3744) [21]. Although its approval is in second line, axitinib has been found to be the most common treatment option, in real-world treatment patterns in the USA, in patients with mRCC progressing after TKI and mTOR [22].

Under this perspective, we hypothesized that axitinib might be an active and safe option for patients with mRCC who receive this treatment later on their treatment sequence. We conducted a retrospective study of patients with mRCC treated in our department that received axitinib beyond second line aiming to study the efficacy and safety of this agent in this subgroup of patients.

## Methods

This is a single-institutional, retrospective study, which was carried out in Hematology-Oncology Unit of Clinical Therapeutics Department of University of Athens in Alexandra General Hospital. Medical records of patients who were treated with axitinib beyond second line, between August 2013 and January 2017 were retrospectively reviewed. All subjects had given written informed consent. Only patients aged above 18 years old that had received at least two treatment lines for metastatic, histologically confirmed RCC, before the initiation of axitinib were recorded. Data was collected through a single institution database consisted of demographic, clinicopahtological and treatment-related and survival data.

### Statistical analysis

Descriptive statistics were used to assess clinicopathological parameters of the patients. OS was calculated from the initiation of axitinib treatment until death or last follow-up. The effect of the expression of the various clinicopathological parameters on clinical outcome was assessed by plotting survival curves according to the Kaplan–Meier method and comparing groups using the log-rank test. Patients with PFS on axitinib treatment > 12 months were considered as having long term benefit from the treatment and were also analyzed separately to identify possible factors related to this response. The associations of each of the above-indicated factors and clinicopathological parameters with OS were assessed through hazard ratios estimated from univariate Cox proportional hazards models. Factors for which the hazard ratios were statistically significant at the level of significance 0.1 were then included simultaneously in a multivariate Cox proportional hazards model using the backward-stepwise method with removal criterion *p* > 0.10. All results with a two-sided p level ≤ 0.05 were considered statistically significant, whereas a *p* value between 0.05 and 0.10 was considered of borderline significance. Statistical analyses were performed using SPSS software package, version 21 (Computing Resource Centre, Santa Monica, California, USA) and GraphPad Prism software (GraphPad Software Inc., La Jolla, California, USA).

## Results

### Clinicopathological characteristics and previous treatments

22 patients initiated Axitinib in our institution as third or further line of treatment for their renal cancer from December 2013 to January 2017 and were recorded in our study. Clinicopathological characteristics of the patients as well as data regarding their previous treatments are presented in Table 1. All patients had undergone radical nephrectomy and the histological diagnosis was predominant Clear Cell Renal cell carcinoma (ccRCC) in 17 (77%) patients, while 5 (23%) patients had non-ccRCC tumors At the beginning of 1st line treatment 20 patients (91%) were evaluable for categorizing into Heng prognostic groups, resulting in 6 (30%), 13 (65%), and 1 (5%), good, intermediate and poor prognostic scores respectively. The majority of patients (12 patients, 54,5%) received axitinib as third line treatment, while 4 patients (18,2%) received drug as fourth line treatment and 6 patients (27,3%) beyond fourth line of treatment. At the beginning of axitinib treatment, 19 patients (85,3%) had already received VEGF-TKIs and mTOR inhibitors as previous lines of therapy, 1 patients (4,5%) VEGF-TKIs only and 2 patients (9,1%) VEGF-TKIs and immune checkpoint inhibitors. Also, 11 patients (50%) had received only one prior VEGF-TKI, while the remaining 11 patients two or more different drugs of this class of agents (Table 2). The duration of treatment with axitinib was 6.15 months (range 3.26–8.2 months). Patients were followed after the initiation of axitinib for a median period of 13.5 months (range 2.6–39.18 months). The majority of patients that started axitinib treatment had significant burden of disease. More specifically, 18 patients (81,8%) had multiple sites of metastases, while 9 patients (40,9%) had liver disease and 5 patients (22,7%) had bone disease. As a result, at the beginning of axitinib treatment, 9 patients were classified as intermediate risk (40,9%) and 11 patients (50%) as poor prognosis according to Heng criteria.

**Table 1:** Clinicopathological and treatment-related characteristics of the patients

Characterstics	Median	Range
**Age**	55,4	30,2-73,5
	N	%
**Sex**		
Male	11	50
Female	11	50
**Histology**		
ccRCC	17	77,3
non-ccRCC	5	22,7
**Nephrectomy**		
Yes	22	100
No	0	0
**Fuhrman grade**		
< 3	6	27,3
≥ 3	13	59,1
NA	3	13,6
**Heng prognostic group at 1st line**		
Good	6	27,3
Intermediate	13	59,1
Poor	1	4,5
NA	2	9,1
**Heng prognostic group at axitinib line**		
Good	0	0
Intermediate	9	40,9
Poor	11	50
NA	2	9,1
**Axitinb line of treatment**		
3	12	54,5
4	4	18,2
> 4	6	27,3
**Previous treatments**		
TKI only	1	4,5
TKI/IO	2	9,1
TKI/mTOR inhibitor	19	85,3
**No of previous TKIs received**		
< 2	11	50
≥ 2	11	50
**No of metastatic sites at axitinib treatment**		
solitary	4	18,2
multiple	18	81,8
**Sites of metastases at axitinib treatment**		
lymph nodes	12	54,5
lung	19	85,3
liver	9	40,9
bone	5	22,7
local relapse	8	36,4

**Table 2:** Treatments before Axitinib

	1st line (*n* = 22)		2nd line (*n* = 22)		3rd line (*n* = 10)		4th line (*n* = 6)		5th line (*n* = 3)	
Agent(s)	n	%	n	%	n	%	n	%	n	%
Sunitinib	14	63.6	0	0	1	10	0	0	0	0
Pazopanib	3	13.6	3	13.6	1	10	4	66.6	1	33.3
Sunitinib- Everolimus (Sequential)	4	18.2	4	18.2	0	0	0	0	0	0
Bevacizumab	1	4.5	0	0	1	10	0	0	0	0
Temsirolimus- Bevacizumab (Concurrent)	0	0	2	9	0	0	0	0	0	0
Nivolumab	0	0	1	4.5	1	10	0	0	0	0
Sorafenib	0	0	1	4.5	3	30	0	0	0	0
Dovitinib	0	0	0	0	1	10	0	0	0	0
Everolimus	0	0	11	50	2	20	2	33.3	0	0
Temsirolimus	0	0	0	0	0	0	0	0	2	66.6
Total	22	100	22	100	10	100	6	100	3	100

### Efficacy results

**Fig. 1:**
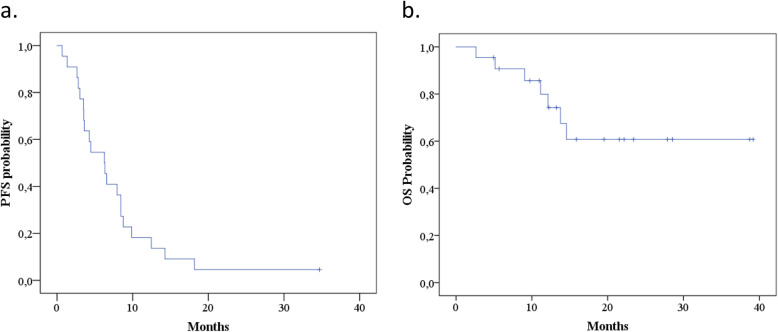
Progression Free Survival (**a**) and Overall Survival (**b**) after axitinib initiation

At the time of data cut off only 1 patient (4,5%) remained on treatment with axitinib. The majority of patients (13/68%) had clinical benefit from axitinib therapy resulting in 6 (27%) partial responses and 9 (41%) stabilizations of disease by RECIST 1.1 criteria, while 7 patients (32%) had disease progression as best response to the treatment. Median PFS was 6.27 months (95% CI 3.62–8.91 months) (Fig. 1a) and median OS after initiation of axitinib treatment was not reached (Fig. 1b). No clinicopathological factor (sex, histology, sites of metastases, Heng criteria) or treatment related factors [line of axitinib treatment (3rd line or beyond 3rd line), number of previous TKIs (1 or ≥ 2), type of previous treatment (TKI only, TKI/mTORi, TKI/IO) and dose reduction] had predictive significance in the univariate analysis (Table 3).

**Table 3:** Univariate analysis of Progression Free Survival

Variables	***N***	mPFS	95% CI	***P*** value
**Sex**				
Male	10	4267	0,000 - 11,085	0,936
Female	11	6267	3318 - 9216	
**Histology**				
Clear Cell	16	4267	2568 - 5965	0,227
Non Clear-Cell	5	8433	7360 - 9507	
**Heng Group**				
Good	5	3.5	2498 - 4502	0,027
Intermediate	13	6.567	1478 - 11,655	
Poor	1	18.167	. .	
**Sites Of Metastasis (Baseline)**				0,738
1	8	4467	1695 - 7239	
2	6	3533	0,000 - 7214	
3	5	3,6	2383 - 4817	
**Sites Of Metastasis (Axitinib)**				
1	4	3033	0,000- 8064	0,963
2	2	3,5	. .	
3	12	4267	2796 - 5738	
4	3	6333	6227–6440	
**Previous TKIs**				
< 2	11	4467	1158 - 7775	0,704
≥ 2	11	6267	3064 - 9469	
**Axitinib line of treatment**				
3	12	4467	0,000 - 9615	0,443
> 3	9	6267	0,423 - 12,110	
**Dose Reduction**				
Yes	5	8433	7360 - 9507	0,216
No	16	3,6	2163 - 5037	
**Type of previous treatment**				
TKI only	1	9866		0,183
TKI/IO	2	2,8		
TKI/mTORi	19	6333	1524-3347	

**Fig. 2:**
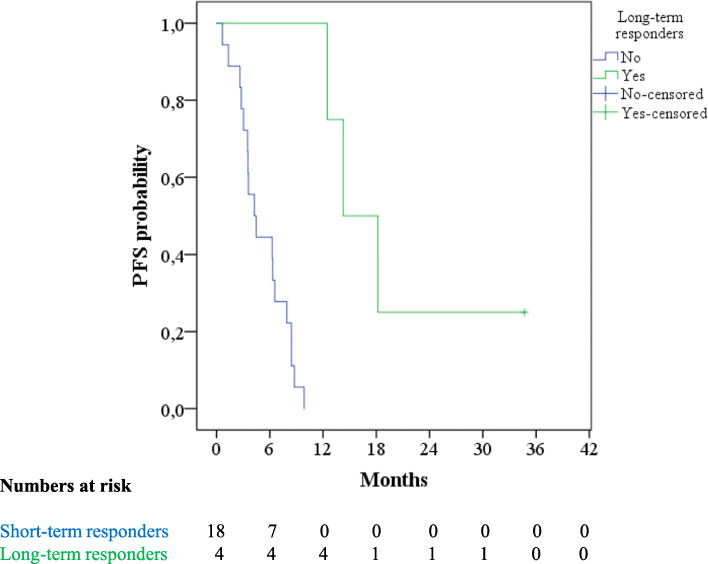
Progression Free Survival in Long-Term responders and the remaining patients in our cohort

Four patients (18.2%) gained long-term benefit from axitinib treatment exceeding 12 months. mPFS in this group of patients was 14.3 months (95% CI 8.7–19.9), while in the remaining patients mPFS was 4.3 months (95% CI 2.5–6.1) (Fig. 2). Two of these patients achieved Partial Response with axitinib treatment, while the other two had Stable Disease. Again, no specific correlation was found between long-term responders and previously described clinicopathological and treatment-related parameters. However, the small number of patients is a limitation of this statistical analysis.

### Safety

Regarding safety, all but one patients (95.8%) experienced adverse events during axitinib treatment (Table 4). The majority of them were grade 1–2 (69%) while ≥ grade 3 toxicities were recorded in 31% of patients. Dose reduction and drug discontinuation had to be performed for 5 (22%) and 3 patients (13%) respectively. The five more common toxicities independently of grade were fatigue (11 patients/50%), hypertension (5 patients/ 23%), hypothyroidism (5 patients/ 23%), hand foot syndrome (HFS) (4 patients/ 18%) and hoarseness (4 patients/ 18%) whereas the two more common grade ≥ 3 toxicities were HFS (4 patients/ 18%) and hypertension (3 patients/ 14%).

**Table 4:** Adverse Events recorded in patients during axitinib treatment

Adverse Event	Any Grade ***N***(%)	Grade >=3 ***N***(%)
Hypertension	5 (22,72)	3 (13,63)
Arthralgia	2 (9,09)	1 (4,54)
Fatigue	11 (50)	1 (4,54)
Hypothyreoidism	5 (22,72)	0 (0)
Increased Liver Function Tests	3 (13,63)	1 (4,54)
Diarrhea	3 (13,63)	0 (0)
Hand Foot Syndrom	4 (18,18)	4 (18,18)
Anorexia	1 (4,54)	1 (4,54)
Mucositis	2 (9,09)	1 (4,54)
Muscle paitn	1 (4,54)	1 (4,54)
Hoarseness	4 (18,18)	1 (4,54)
Proteinurua	2 (9,09)	0 (0)
Anemia	2 (9,09)	0 (0)

## Discussion

Renal cell carcinoma is a common malignancy with many treatment options developed during the last decade. Targeting VEGF and PD-L1 signaling have enriched the armamentarium of the therapeutic options and have prolonged both OS and PFS [5, 6, 8, 9, 13]. However, the existence of several TKIs and the addition of Immunotherapy (IO) resulted to a more complex therapeutic algorithm in the treatment of metastatic RCC [13]. Further it should be noted that TKIs are still studied. Clinical trial NCT02330783 studies the combination of sorafenib and bevacizumab in third line treatment of mRCC. Clinical trial NCT02627963 compares tivozanib and sorafenib in third or fourth line of treatment of patients with mRCC.

Under this perspective data regarding the efficacy of TKIs in the later lines of therapy are important. This is also the case for axitinib that lacks randomized data for efficacy beyond second line and very few published data exist regarding its administration beyond second line. Our study aimed at providing efficacy and safety data for axitinib in this setting. These data provide evidence that axitinib is an active agent even in latter lines of therapy in mRCC as objective responses and mPFS is favorably compared to those achieved in second line in the AXIS trial. Our data are also in agreement with other case series presented in the literature. A large retrospective study with real world data from the United States, regarding axitinib administration resulted that axitinib was most commonly prescribed as a third line treatment [23], while another cohort of patients that received axitinib as either a 2nd line or 3rd line treatment, showed similar activity and tolerability with clinical trials [24]. The efficacy of axitinib beyond second line in our study prompt us to further investigate possible clinicopathological or treatment-related factors that could influence this result. However, no predictive factor was indicated by the analysis, possibly due to the small number of patients. We should also take into consideration that the population of patients that receive treatment beyond second line is usually already selected for improved survival.

The role of axitinib beyond second line was studied retrospectively in a study of Mclean et al. using linked datasets from 22 specialty pharmacies that dispense axitinib with databases of longitudinal medical and pharmacy claims [23]. In this study axitinib was prescribed as third-line therapy in 326 patients and as fourth-line or later therapy in 190 patients resulting in a median duration of treatment of 122 (7–644) and 111 days (10–642). In another retrospective study of third line targeted treatment among 1012 patients with mRCC treated at 25 centers from all over the world axitinib was given in 90 patients resulting in 5.9 months PFS (4.6–8.0), 19.2 months OS (13.5–22.7) and 66% RR [25].

Finally in another retrospective analysis of the data of patients participated in NCT00835978 and NCT00920816 studies revealed a subgroup of long- term responders whereas the duration of treatment with axitinib -as a second line treatment- have been found to be associated with increased frequency of early tumor shrinkage, greater magnitude of tumor shrinkage, and a favorable OS [26].

An important finding of our study was though that four out of the 22 patients had clinical benefit from the axitinib treatment for more than a year. Again previous therapeutic options or other clinicopathological data included to our study, failed to prove any predictive value. This kind of analysis, even though it is retrospective and includes a limited number of patients, is a unique attempt to discover predictive factors; in order to choose the responsive subgroup of patients with mRCC to axitinb treatment, beyond second line. A retrospective analysis of two clinical trials with patients with mRCC who received axitinib as a first line treatment, revealed a population of 37.8% who were long responders [26]. This response was related ECOG performance status 0, no liver or bone metastases, favorable hematology characteristics and baseline tumor burden below the overall median [26].

The molecular pathway that drives this response to axitinib is unknown. Long-term responses have also been described to other TKIs. Long term response to sunitinib has been studied retrospectively from several authors concluding that the absence of adverse disease characteristics (poor PS, poor MSKCC risk, absence of bone or liver metastases) may predict long responders [27–29]. Analogous results have been presented for Pazopanib [30, 31] and Sorafenib [32], but in no case they could provide clinical guidance for the optimal use of these agents in latter lines of therapy.

No new safety signals were recognized in our cohort of patients. Toxicity profile, dose reductions and treatment discontinuation of our patients was similar to the registrational trial of axitinib as a second line treatment [20]. The most frequent adverse events occurring in more than 30% of patients in Axis study; associated with axitinib were diarrhea, hypertension, fatigue, decreased appetite, nausea, and dysphonia, while grade 3 or higher toxicity included hypertension, diarrhea and fatigue [20]. The five most common toxicities independently of grade in our analysis were fatigue, hypertension, hypothyroidism, hand foot syndrome and hoarseness, with grade 3 or higher toxicities including hypertension and hand foot syndrome. It is evident that axitinib administration beyond second line, even in heavily pretreated patients; is not accompanied by a more severe pattern of adverse effects.

We must highlight the caveats of our study, which is a retrospective non randomized study with a small number of patients included. Several confounding factors such as selection bias, heterogeneous treatments prior to axitinib and a relatively short period of follow up are crucial limitations that should be reported. Further, this analysis is based only to clinicopathological data without any translational studies and assessment of quality of life to accompany and support the clinical outcome.

In conclusion, axitinib is an effective treatment option for patients with mRCC beyond second line. Axitinib is well tolerated from patients, even heavily pretreated. Further studies including larger numbers of patients, combined with translational research analyses; are needed to discover the subgroup of patients who are responsive to this treatment beyond second line.

## Conclusions

Antiangiogenic targeted therapy still remains an important element of RCC treatment. Axitinib is a tyrosine kinase inhibitor with proven efficacy in RCC as a 2nd line treatment. Our retrospective study highlights Axitinib’s efficacy and safety in third and further line treatment in RCC.

## Data Availability

The datasets generated and analyzed during the current study are not publicly available due to restriction of the ethics committee of General Hospital Alexandra, Athens, Greece, but are available from the corresponding author on reasonable request.

## Abbreviations

mRCC: Metastatic renal cell carcinoma
mTOR: *Mammalian target of rapamycin*
VEGF: Vascular endothelial growth factor
TKI: Tyrosine kinase inhibitor
EAU: European association of urology
OS: Overall survival
PFS: Progression free survival
PS: Performance status
ECOG: Eastern Cooperative Oncology Group
MSKCC: Memorial Sloan kettering cancer center

## References

[1] Zagouri F, Peroukidis S, Tzannis K, Kouloulias V, Bamias A, Hellenic Genito-Urinary Cancer G (2015). Current clinical practice guidelines on chemotherapy and radiotherapy for the treatment of non-metastatic muscle-invasive urothelial cancer: a systematic review and critical evaluation by the Hellenic Genito-urinary Cancer group (HGUCG). Crit Rev Oncol Hematol.

[2] Bamias A, Escudier B, Sternberg CN, Zagouri F, Dellis A, Djavan B, Tzannis K, Kontovinis L, Stravodimos K, Papatsoris A (2017). Current clinical practice guidelines for the treatment of renal cell carcinoma: a systematic review and critical evaluation. Oncologist.

[3] Cohen HT, McGovern FJ (2005). Renal-cell carcinoma. N Engl J Med.

[4] Shingarev R, Jaimes EA (2017). Renal cell carcinoma: new insights and challenges for a clinician scientist. Am J Physiol Renal Physiol.

[5] Motzer RJ, Hutson TE, Tomczak P, Michaelson MD, Bukowski RM, Rixe O, Oudard S, Negrier S, Szczylik C, Kim ST (2007). Sunitinib versus interferon alfa in metastatic renal-cell carcinoma. N Engl J Med.

[6] Sternberg CN, Davis ID, Mardiak J, Szczylik C, Lee E, Wagstaff J, Barrios CH, Salman P, Gladkov OA, Kavina A (2010). Pazopanib in locally advanced or metastatic renal cell carcinoma: results of a randomized phase III trial. J Clin Oncol.

[7] Escudier B, Eisen T, Stadler WM, Szczylik C, Oudard S, Siebels M, Negrier S, Chevreau C, Solska E, Desai AA (2007). Sorafenib in advanced clear-cell renal-cell carcinoma. N Engl J Med.

[8] Escudier B, Pluzanska A, Koralewski P, Ravaud A, Bracarda S, Szczylik C, Chevreau C, Filipek M, Melichar B, Bajetta E (2007). Bevacizumab plus interferon alfa-2a for treatment of metastatic renal cell carcinoma: a randomised, double-blind phase III trial. Lancet.

[9] Choueiri TK, Escudier B, Powles T, Mainwaring PN, Rini BI, Donskov F, Hammers H, Hutson TE, Lee JL, Peltola K (2015). Cabozantinib versus Everolimus in advanced renal-cell carcinoma. N Engl J Med.

[10] Motzer RJ, Escudier B, Oudard S, Hutson TE, Porta C, Bracarda S, Grunwald V, Thompson JA, Figlin RA, Hollaender N (2008). Efficacy of everolimus in advanced renal cell carcinoma: a double-blind, randomised, placebo-controlled phase III trial. Lancet.

[11] Hudes G, Carducci M, Tomczak P, Dutcher J, Figlin R, Kapoor A, Staroslawska E, Sosman J, McDermott D, Bodrogi I (2007). Temsirolimus, interferon alfa, or both for advanced renal-cell carcinoma. N Engl J Med.

[12] Motzer RJ, Hutson TE, Glen H, Michaelson MD, Molina A, Eisen T, Jassem J, Zolnierek J, Maroto JP, Mellado B (2015). Lenvatinib, everolimus, and the combination in patients with metastatic renal cell carcinoma: a randomised, phase 2, open-label, multicentre trial. Lancet Oncol.

[13] Motzer RJ, Escudier B, McDermott DF, George S, Hammers HJ, Srinivas S, Tykodi SS, Sosman JA, Procopio G, Plimack ER (2015). Nivolumab versus Everolimus in advanced renal-cell carcinoma. N Engl J Med.

[14] Fogli S, Porta C, Del Re M, Crucitta S, Gianfilippo G, Danesi R, Rini BI, Schmidinger M (2020). Optimizing treatment of renal cell carcinoma with VEGFR-TKIs: a comparison of clinical pharmacology and drug-drug interactions of anti-angiogenic drugs. Cancer Treat Rev.

[15] Gulati S, Vaishampayan U (2020). Current state of systemic therapies for advanced renal cell carcinoma. Curr Oncol Rep.

[16] Angulo JC, Shapiro O. The Changing Therapeutic Landscape of Metastatic Renal Cancer. Cancers (Basel). 2019;11(9):1227. 10.3390/cancers11091227 PMC6770566 31443471

[17] Ljungberg B, Bensalah K, Canfield S, Dabestani S, Hofmann F, Hora M, Kuczyk MA, Lam T, Marconi L, Merseburger AS (2015). EAU guidelines on renal cell carcinoma: 2014 update. Eur Urol.

[18] Motzer RJ, Porta C, Vogelzang NJ, Sternberg CN, Szczylik C, Zolnierek J, Kollmannsberger C, Rha SY, Bjarnason GA, Melichar B (2014). Dovitinib versus sorafenib for third-line targeted treatment of patients with metastatic renal cell carcinoma: an open-label, randomised phase 3 trial. Lancet Oncol.

[19] Calvo E, Escudier B, Motzer RJ, Oudard S, Hutson TE, Porta C, Bracarda S, Grunwald V, Thompson JA, Ravaud A (2012). Everolimus in metastatic renal cell carcinoma: subgroup analysis of patients with 1 or 2 previous vascular endothelial growth factor receptor-tyrosine kinase inhibitor therapies enrolled in the phase III RECORD-1 study. Eur J Cancer.

[20] Rini BI, Escudier B, Tomczak P, Kaprin A, Szczylik C, Hutson TE, Michaelson MD, Gorbunova VA, Gore ME, Rusakov IG (2011). Comparative effectiveness of axitinib versus sorafenib in advanced renal cell carcinoma (AXIS): a randomised phase 3 trial. Lancet.

[21] Motzer RJ, Escudier B, Tomczak P, Hutson TE, Michaelson MD, Negrier S, Oudard S, Gore ME, Tarazi J, Hariharan S (2013). Axitinib versus sorafenib as second-line treatment for advanced renal cell carcinoma: overall survival analysis and updated results from a randomised phase 3 trial. Lancet Oncol.

[22] Pal SK, Signorovitch JE, Li N, Zichlin ML, Liu Z, Ghate SR, Perez JR, Vogelzang NJ (2017). Patterns of care among patients receiving sequential targeted therapies for advanced renal cell carcinoma: a retrospective chart review in the USA. Int J Urol.

[23] MacLean E, Cisar L, Mehle K, Eremina D, Quigley JM (2016). Real-World Axitinib Use in the United States: A Retrospective Study Using Linked Datasets. J Manag Care Spec Pharm.

[24] Hutson TE, Jiao X, Wilson T, Cisar L, MacLean EA (2017). Axitinib in metastatic renal cell carcinoma: patient characteristics and treatment patterns in US community oncology centers. Future Oncol.

[25] Kyriakopoulos CE, Chittoria N, Choueiri TK, Kroeger N, Lee JL, Srinivas S, Knox JJ, Bjarnason GA, Ernst SD, Wood LA (2015). Outcome of patients with metastatic sarcomatoid renal cell carcinoma: results from the international metastatic renal cell carcinoma database consortium. Clin Genitourin Cancer.

[26] Rini BI, Gruenwald V, Jonasch E, Fishman MN, Tomita Y, Michaelson MD, Tarazi J, Cisar L, Hariharan S, Bair AH (2017). Long-term duration of first-line Axitinib treatment in advanced renal cell carcinoma. Target Oncol.

[27] Molina AM, Jia X, Feldman DR, Hsieh JJ, Ginsberg MS, Velasco S, Patil S, Motzer RJ (2013). Long-term response to sunitinib therapy for metastatic renal cell carcinoma. Clin Genitourin Cancer.

[28] Smaletz O, Chacon M, de Oliveira KL, de Carvalho Rocha DR, Cardoso FC (2016). Long-term benefit of sunitinib in patients with metastatic renal cell carcinoma in Latin America: retrospective analysis of patient clinical characteristics. Onco Targets Ther.

[29] Tannir NM, Figlin RA, Gore ME, Michaelson MD, Motzer RJ, Porta C, Rini BI, Hoang C, Lin X, Escudier B. Long-term response to Sunitinib treatment in metastatic renal cell carcinoma: a pooled analysis of clinical trials. Clin Genitourin Cancer. 2017;S1558-7673(17)30171–4. 10.1016/j.clgc.2017.06.005 PMC6736765 28711490

[30] Park J, Jiao X, Ghate S, Wilson T, Ahmad QI, Vogelzang NJ. Predictors of long-term response with Pazopanib in patients with advanced renal-cell carcinoma. Clin Genitourin Cancer. 2018;16(4):293–7. 10.1016/j.clgc.2018.03.005 29653813

[31] Sbrana A, Biasco E, Paolieri F, Palesandro E, Caserta C, Iacovelli R, Detti B, Santini D, Mosca A, Morelli F (2018). Long-term response to first-line Pazopanib therapy in mRCC patients: a multicenter Italian experience. Anticancer Res.

[32] Zhang HL, Qin XJ, Wang HK, Gu WJ, Ma CG, Shi GH, Zhou LP, Ye DW (2015). Clinicopathological and prognostic factors for long-term survival in Chinese patients with metastatic renal cell carcinoma treated with sorafenib: a single-center retrospective study. Oncotarget.

